# Robotic hand illusion with tactile feedback: Unravelling the relative contribution of visuotactile and visuomotor input to the representation of body parts in space

**DOI:** 10.1371/journal.pone.0210058

**Published:** 2019-01-23

**Authors:** The Vu Huynh, Robin Bekrater-Bodmann, Jakob Fröhner, Joachim Vogt, Philipp Beckerle

**Affiliations:** 1 Work and Engineering Psychology Research Group, Technische Universität Darmstadt, Darmstadt, Germany; 2 Department of Cognitive and Clinical Neuroscience, Central Institute of Mental Health, Medical Faculty Mannheim, Heidelberg University, Mannheim, Germany; 3 Chair of Information-oriented Control, Department of Electrical and Computer Engineering, Technical University of Munich, Munich, Germany; 4 Elastic Lightweight Robotics Group, Robotics Research Institute, Department of Electrical Engineering and Information Technology, Technische Universität Dortmund, Dortmund, Germany; 5 Institute for Mechatronic Systems in Mechanical Engineering, Technische Universität Darmstadt, Darmstadt, Germany; Anglia Ruskin University, UNITED KINGDOM

## Abstract

The rubber hand illusion describes a phenomenon in which participants experience a rubber hand as being part of their body by the synchronous application of visuotactile stimulation to the real and the artificial limb. In the recently introduced robotic hand illusion (RobHI), a robotic hand is incorporated into one’s body representation due to the integration of synchronous visuomotor information. However, there are no setups so far that combine visuotactile and visuomotor feedback, which is expected to unravel mechanisms that cannot be detected in experimental designs applying this information in isolation. We developed a robotic hand, controlled by a sensor glove and equipped with pressure sensors, and varied systematically and separately the synchrony for motor feedback (MF) and tactile feedback (TF). In Experiment 1, we implemented a ball-grasping task and assessed the perceived proprioceptive drift of one’s own hand as a behavioral measure of the spatial calibration of body coordinates as well as explicit embodiment experiences by a questionnaire. Results revealed significant main effects of both MF and TF for proprioceptive drift data, but we only observed main effects for MF on perceived embodiment. Furthermore, for the proprioceptive drift we found that synchronous feedback in one factor compensates for asynchronous feedback in the other. In Experiment 2, including a new sample of naïve participants, we further explored this finding by adding unimodal conditions, in which we manipulated the presence or absence of MF and/or TF. These findings replicated the results from Experiment 1 and we further found evidence for a supper-additive multisensory effect on spatial body representation caused by the presence of both factors. Results on conscious body perception were less consistent across both experiments. The findings indicate that sensory and motor input equally contribute to the representation of spatial body coordinates which for their part are subject to multisensory enhancing effects. The results outline the potential of human-in-the-loop approaches and might have important implications for clinical applications such as for the future design of robotic prostheses.

## Introduction

The perception of one’s own body parts requires the simultaneous processing and combination of a variety of sensorimotor signals which contribute to a coherent representation of the body [1]. Previous research indicates that the body representation is inextricably linked to the representation of the close surrounding of the body, i.e., the peripersonal space (e.g., [2]), potentially facilitating the discrimination between the self and the environment. This discrimination is necessary for any successful movement in or interaction with the surroundings [3].

The flexibility of the peripersonal space is closely related to the plasticity of body representation [4]. The so-called rubber hand illusion (RHI; [5]) offers the opportunity to experimentally manipulate this continuous process. In this experiment, a life-like rubber hand is placed in front of a participant while the participant’s real hand is hidden from view. When the rubber hand is visually stimulated in synchrony with touches applied to the real but hidden hand, the majority of participants report to feel the touch in the artificial limb, accompanied by the sensation of ownership for this hand, i.e., the perception that the artificial limb belongs to the stimulated individual [6]. Asynchronous stimulation, however, dissolves the illusory sensation. As a behavioral proxy of successful RHI induction, the own hand is perceived to be closer to the rubber hand than before illusion induction. Since this effect (often referred to as proprioceptive drift) is associated with the duration of illusion induction [5] as well as the intensity of illusory sensations [7], it has often been interpreted as a consequence of a multimodal recalibration process of the limb’s proprioceptive representation in peripersonal space.

In the last two decades, several variations of the original RHI setup have been developed. Besides rubber limbs, researchers showed that mirrored limbs (e.g., [8]), limbs in virtual reality (e.g., [9]), or robotic hands (e.g., [10]) can be induced to be perceived as belonging to the participants’ body by visuotactile stimulation. The communality of these studies is a passive setup, which admittedly enables the accurate application of visuotactile stimulation but explicitly excludes movements, which per se represent an important information source for the calibration of spatial body coordinates and body perception. Thus, using more recent setups, it has been shown that both active and passive movements induce equally strong ownership sensations for an artificial limb [11], but only active, compared to passive, movements appeared to calibrate the spatial representation of the body as a whole, as indicated by a proprioceptive drift spread across body parts [12]. This effect has been related to the induction of agency (i.e., the sensation that somebody is the initiator of certain actions; [6]), a component which has also been reported to be induced in the original static RHI setup [7], albeit weaker in extent. Analogous to the static RHI, asynchronous visuomotor information has been used as control condition in this moving RHI (e.g., [13]). Paradigms based on the moving RHI also have been translated into other contexts, such as virtual reality [14]. Another context is the field of robotics: in the so-called robotic hand illusion (RobHI) paradigm, participants control a robotic hand according to their own hand’s movements [15–17]. As in the moving RHI, participants in the RobHI report a sense of agency, accompanied by a proprioceptive drift [15]. This kind of setup not only enables to feedback movements with very low intrinsic delays, but further offers the opportunity to precisely implement delayed motor feedback as control conditions (e.g., [18]).

Only few studies so far, however, have implemented both visuotactile and visuomotor feedback in one single setup (e.g., [19, 20]) and–to our best knowledge–no study experimentally manipulated both visuomotor and visuotactile congruency simultaneously in order to assess effects on explicit and implicit body representations. Advancements in robotics could potentially close this critical gap, and could further enhance external validity of experimental designs on the representation of the body in space and its associated perceptions.

In the present study, we equipped a robotic hand (as described in [16]) with tactile feedback (as described in [20, 21]), which in contrast to previous RobHI setups not only allows for the precise control of visuomotor feedback (immediate or delayed) but also enables us to manipulate independently the timing of tactile feedback (also immediate or delayed). In two experiments, using full-factorial designs each, we thus wanted to answer the question how the independent manipulation of synchrony between a) visual and tactile input and b) visual and motor information as well as c) their interplay influence body representation by assessing explicit questionnaire measures and the implicitly perceived location of one’s own hand, indicative for shifts of body coordinates in peripersonal space. In Experiment 1, we systematically varied the synchrony (i.e., factor levels *synchronous* and *asynchronous*) of both motor and tactile feedback independently and evaluate the effects on implicit and explicit measures of the RobHI. In Experiment 2, we additionally added the factor level *absence* for both kinds of feedback, enabling us to further elucidate the relative contribution of sensorimotor feedback to spatial body representation and conscious body perception.

These results might have important implications for the understanding of multimodal mechanisms behind the representation of the body and the peripersonal space. Furthermore, this study outlines how human-in-the-loop approaches [21, 22] can facilitate the experimental modification and decomposition of effects that are inseparably connected in humans. In contrast to virtual reality studies, human-in-the-loop experiments might directly guide the design of prosthetic devices and other assistive robotics due to investigating how people respond to physically real hardware.

## Methods

### Participants

In total, we recruited 62 participants. Forty-four participants (23 females; mean age = 21.02 years, standard deviation (SD) = 2.33) were included in Experiment 1, whereas 18 (7 females; mean age = 22.67 years, SD = 2.03) newly recruited and naïve participants took part in Experiment 2. Sample sizes for Experiment 1 were selected based on (a priori) expected small effect sizes, while sample sizes for Experiment 2 were based on the actually large effect sizes observed in the first experiment. The majority of participants were students of the Technische Universität Darmstadt. None of them reported experiences with the original RHI setup in the past. Prior to participating in the experiments, participants provided their written informed consent. This study was conducted with a positive vote by the ethics committee of the Technische Universität Darmstadt (reference number: EK23/2016) and is in accordance with the Declaration of Helsinki in its current version.

### Apparatus

**Fig 1:**
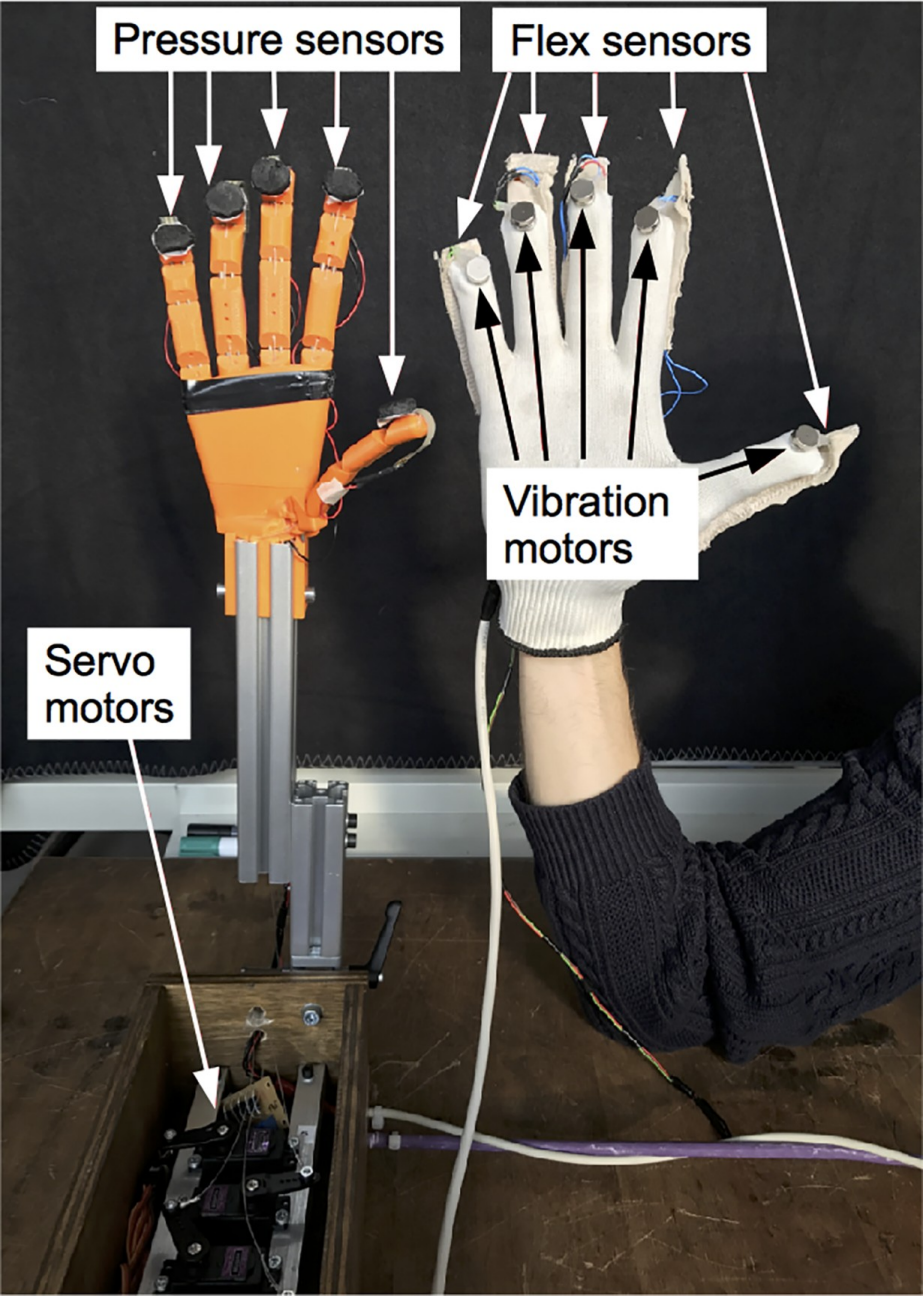
Sensor glove and robotic hand without protective gloves.

The experimental setup consisted of a custom-made robotic hand and a sensor glove (Fig 1) which were designed and implemented based on previous concepts [16, 23]. The single digits of the robotic hand were 3D-printed acrylonitrile butadiene styrene elements which were connected by steel springs, mimicking the joints of the hand. In order to increase the methodological value of the robotic hand, pressure sensors (FSR 402, Interlink Electronics Inc., Westlake Village, California, USA) at its fingertips were used to acquire contact pressure which was mapped to vibration motors (VPM2, Precision Microdrives, London, United Kingdom) at the fingertips of the glove worn by the participants. Vibrotactile stimulation has previously been used to successfully induce the RHI [24] and a virtual hand illusion [19, 25]. The force sensitivity range of the pressure sensors was approximately between 0.2 and 20 N. Along the fingers of the sensor glove, we attached flex sensors (FS, Spectra Symbol, Salt Lake City, Utah, USA) which measure the flexion of the fingers at a sampling rate of 50 Hz. These values were mapped to servo motors (MG995R, Tower Pro Pte Ltd, Taiwan) which pulled nylon cords connected to the fingertips of the robotic hand and thus moved them accordingly to the real hand’s finger movements with a measured latency of approximately 120 ms (i.e., the system-intrinsic delay). In the same way, the delay of the vibration mapping was measured and resulted in an averaged delay of about 80 ms. Therefore, the asynchronous condition consisted of an overall delay of 620 ms, i.e., the sum of the system intrinsic delay of 120 ms and the artificial delay of 500 ms. The system was controlled by a microcontroller (Mega2560 R3 ATmega2560-16AU, SainSmart, Lenexa, Kansas, USA). For the present study, the robotic hand and the sensor glove were customized and were programmed to provide specified delays in motor and tactile feedback. Therefore, a timer was implemented to add the desired feedback in the asynchronous conditions. We chose a delay of 500 ms for both the tactile and the movement feedback, since this value exceeds what has been shown to significantly reduce illusory experiences in previous studies applying visuotactile [9, 25] and visuomotor asynchrony [18, 26, 27]. An additional timer was set to stop each condition after three minutes in a controlled manner.

### Procedure

**Fig 2:**
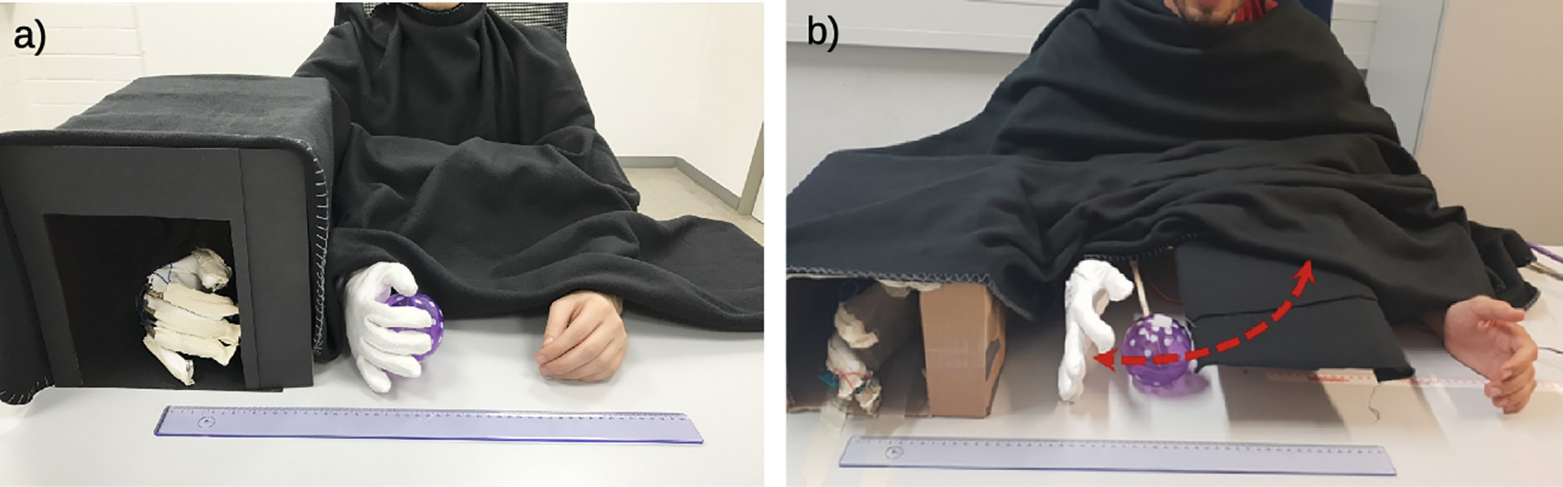
Experimental setups. (a) Experiment 1: The right hand of the participant is placed inside a box and equipped with a sensor glove. The box and the participant’s upper body are covered by a black cloth. The participant is instructed to grasp and release the ball. (b) Experiment 2: A ball device is attached to the robotic hand system. When the ball is at its zero position (maximally away from the open robotic hand), it is hidden under the visual cover. While the robotic hand is closing, the ball moves towards the palm in a circular trajectory (dashed red arrow).

The participants were seated in front of a table and their left hands were placed next to the robotic hand on the table top. Participants were instructed not to move their arm and hand throughout the experiment, apart from performing the grasping movements. The right hand was equipped with the sensor glove and was hidden from view under a box with a distance to the robotic hand of 21 cm, which is within the spatial limits for eliciting the RHI [28, 29]. The box and the upper body of the participant, starting from his or her neck up to the hands including the robotic arm, were covered under a black cloth (Fig 2A).

#### Experiment 1

For implementing a task that includes tactile feedback, we attached a rubber ball (diameter of 6.8 cm) to the robotic hand, which the participants were asked to grasp continuously in a frequency of about 1 Hz. However, not every pressure sensor responded in a similar way; we tackled this problem by modifying the program so that vibration was equally applied to each finger when all fingers were bent and when at least one finger received a pressure exceeding the force caused by the rubber gloves on the robotic hand.

The implementation of delays in the tactile feedback was realized by a loop function which repeatedly read the values from the sensors and transmitted them to the respective motor. When the delay of 500 ms was reached, the values were written to the servo and/or vibration motors until the next cycle started. This resulted in the hand moving or the vibration motors vibrating 500 ms after the sensors had received the signals, with the same duration of the incoming signals.

Before the experiment started, participants were asked to give an initial estimation of the perceived location of their hand. To this end, they were instructed to close their eyes and to point with their left index finger on top of their right hand’s back without touching it. The felt location was then noted as the baseline value for the proprioceptive drift and marked with a sticker. For each measurement, a ruler was aligned to the indicated position, out of the participants’ sight. After each condition (described below), this procedure was repeated and the difference between this value and the baseline was calculated, which represents the proprioceptive drift measurement (in cm).

Experiment 1 consisted of four conditions, in which the factors *synchrony of tactile feedback* (synchronous, i.e., *TFs*, vs asynchronous, i.e., *TFa*) and *synchrony of motor feedback* (likewise synchronous, i.e., *MFs*, vs asynchronous, i.e., *MFa*) were manipulated. In order to evenly and randomly distribute the order of conditions to the participants and to prevent sequence effects, a Latin square with the size of 4 × 4 was used, where the *n*^th^ person was assigned to the (*n* mod 4)^th^ row.

During Experiment 1, the task was to grasp and release the rubber ball with the robotic hand. The participants were instructed to open and close their right hands in order to grasp the ball with a frequency of approximately 1 Hz, which was verified throughout the condition by the experimenter. When the pressure sensor at the fingertips of the robotic hand touched the ball with an above-threshold force, the participant received a tactile feedback through the vibration motors. To ensure the distinctiveness of the conditions, the participants were instructed to wait until they felt the vibration before re-opening the hand. Throughout the experiment, the participants were instructed to observe the robotic hand. They wore earplugs and ear protectors in order to cancel the noise of the motors driving the motions of the robotic hand. Each condition had a duration of 180 seconds and was concluded by the measurement of the proprioceptive drift and a questionnaire containing items introduced previously [7], translated to German (Table 1), aiming at the factors *Ownership*, *Location*, and *Agency*. We used a 5-point Likert scale ranging from −2 (‘strongly disagree’) to +2 (‘strongly agree’).

**Table 1:** Questionnaire items divided into the factors ‘Ownership’ (items 1–5), ‘Location’ (items 6 and 7), and ‘Agency’ (items 8 and 9).

Item	During the block…	Factor
1	… it seemed like I was looking directly at my own hand, rather than a robotic hand.	Ownership
2	… it seemed like the robotic hand began to resemble my real hand.	Ownership
3	… it seemed like the robotic hand belonged to me.	Ownership
4	… it seemed like the robotic hand was my hand.	Ownership
5	… it seemed like the robotic hand was part of my body.	Ownership
6	… it seemed like my hand was in the position where the robotic hand was.	Location
7	… it seemed like the robotic hand was in the position where my hand was.	Location
8	… it seemed like I could have moved the robotic hand if I had wanted.	Agency
9	. . . it seemed like I was in control of the robotic hand.	Agency

#### Experiment 2

Experiment 1 systematically varied the level of synchrony of tactile and motor feedback. However, the combination of synchronous and asynchronous modalities could mask the nature of their interaction. In order to explore spatial and perceptual RobHI effects under unimodal tactile and motor feedback, we devised an additional Experiment 2. Data from unimodal conditions could be relevant for the understanding of the 'mixed' conditions in Experiment 1: is, for example, an asynchronous modality ignored, in which case the effect should be similar to unimodal synchronous feedback, or does it interfere with the other modality, potentially reducing the enhancing effect of sensorimotor combination, which is assumed to maximize information delivered from the different modalities?

In order to answer these questions and to further evaluate the validity of the results obtained in Experiment 1, we again used the robotic hand setup introduced above, and modified it in a way that allowed us to implement synchronous, asynchronous, and unimodal conditions. The ball, which has been placed in the palm of the robotic hand in Experiment 1, has now been mounted to a lever (26.5 cm in length), which was attached to an additional servo motor whose function was coupled to the robotic hand’s movements (Fig 2B): when the robotic hand started to grasp in response to the real hand’s movements, the servo motor caused the lever holding the ball on its distal end to move towards the robotic hand in a circular trajectory. The timing was set in such a way that the ball reached the palm exactly when the robotic hand was closed, independently of whether the robotic hand responded with or without a delay compared to the real hand’s movements. The servo motor, the lever, and most of the ball’s trajectory were hidden under a visual cover in order to not distract the participant by its movements. The ball became visible only just before it reached the robotic hand.

In Experiment 2, we implemented a 3 (*TFs/TFa/TFn*) × 3 (*MFs/MFa/MFn*) within-subjects design (the *n* abbreviates ‘no feedback’ and indicates the absence of the respective modality), resulting in nine conditions, and respectively, a 9 × 9 Latin square for the order of conditions was applied. The adjusted setup enabled us to deliver unimodal TF conditions (*MFn/TFs* and *MFn/TFa*), in which the participants were asked to hold their hand in a slightly opened posture, with the robotic hand having the same posture. The ball could thus stimulate the pressure sensors of the robotic hand’s fingers in a randomized frequency of about 0.8, 1.0 and 1.2 Hz, triggering the tactile feedback without any self-executed movement. Randomization of the frequencies was implemented in order to decrease expectation effects and to mimic the unsteady rhythm of self-paced movements in the other conditions. In order to ensure that the participants did not move their real hand during the *MFn* conditions, we read out the protocol of sensory glove data post-hoc and found no substantial movement caused by the participants’ real hand’s movements. The duration of conditions was identical to Experiment 1. In order to get a more valid estimation of the proprioceptive drift, we asked the participants to blindly indicate the real hand’s position three times each [5], and report on the averaged values. After each condition, we asked participants for their subjective illusory embodiment experiences by using the same questionnaire as described for Experiment 1.

### Statistical analysis

In order to investigate the drift of the perceived hand position towards the robotic hand in both experiments, we performed one sample *t*-tests with the test value 0 separately for each condition and adjusted the *p*-values using Bonferroni correction by multiplying them by the number of tests, i.e., the alpha level in all analyses is .05 throughout the manuscript. Afterwards, we performed separate two-way repeated measures analyses of variance (ANOVAs) with the two factors *tactile feedback* (TF) and *motor feedback* (MF), each with the levels *s* and *a* (synchronous and asynchronous) for Experiment 1, while adding a third level *n* (no feedback, i.e., the absence of the respective modality) for each factor in Experiment 2. If Mauchly’s test became significant (in any of the ANOVAs), indicative of violating the assumption of sphericity, we adjusted values using the Greenhouse-Geisser correction. Main effects, interaction effects, as well as their effect sizes (η^*2*^) are reported. In order to further investigate the mean differences between the conditions, additional post-hoc comparisons (all tests were performed two-tailed) with Bonferroni-corrected *p*-values were used for proprioceptive drift data, and we report test statistics and effect sizes (Cohen’s *d*). We applied the same variance analytic approach to the questionnaire data, which were averaged for each condition and participant. After performing the ANOVA with the averaged questionnaire scores, which was done to give a general impression of perception, the items were separated according to the factors *Ownership*, *Location*, and *Agency* and three two-way repeated measures ANOVAs were performed. Due to similar results for averaged and separated questionnaire data (see results for Experiment 1), we abstained from doing the follow-up analyses for questionnaire factors in Experiment 2.

In order to test whether there is a sensorimotor combination effect on the proprioceptive drift, we calculated a compound score composed of a) the drift in the fully asynchronous condition (*MFa/TFa*) plus b) the supplemental drift under synchronous motor feedback (*MFs/TFa* minus *MFa/TFa*) plus c) the supplemental drift under synchronous tactile feedback *(MFa/TFs* minus *MFa/TFa*). Thus, this score reflected the linear combination of drift under the assumption that both modalities influence this variable independently. If this composed score wouldn’t be significantly different from the data obtained in the fully synchronous condition (i.e., *MFs/TFs*), this would indicate compensation of the detrimental effects of one asynchronous factor by a synchronous one. If the fully synchronous condition would induce a significantly stronger drift, however, this would indicate a super-additive effect caused by sensorimotor combination. We analysed the data from Experiment 2 accordingly, i.e., we compared the compound score described above (replication of Experiment 1) and further compared an analogue compound score for the presence/absence of modalities ((*MFn/TFn* plus (*MFs/TFn* minus *MFn/TFn*) plus (*MFs/TFn* minus *MFn/TFn*)) with the data obtained in the fully synchronous condition. In order to also interpret non-significant differences [30], we applied Bayesian *t*-tests for these analyses and provide the Bayes factor (*BF*_10_). All analyses were performed using JASP [31].

## Results

### Experiment 1

#### Proprioceptive drift

We found significant drifts of perceived hand position towards the robotic hand in each condition (Table 2) by using one-sample *t*-tests comparing the means against the test value 0 (*t*_43_ between 6.24 and 14.26, all *p*_*corr*_ < .001, Cohen’s *d* between 0.94 and 2.15). The repeated measures ANOVA showed a significant main effect for both *MF* (*F*_1,43_ = 168.18, *p* < .001, η^*2*^ = .80) and *TF* (*F*_1,43_ = 104.99, *p* < .001, η^*2*^ = .71), but no significant interaction (*F*_1,43_ = 1.04, *p* = .315, η^*2*^ = .02). These results indicate that both factors individually influence perceived hand position without interacting with each other. Bonferroni-adjusted post-hoc analysis revealed a significant difference (*p*_corr_ < .001 each) in proprioceptive drift measures in the synchronous compared to the asynchronous levels in both modalities.

**Table 2:** Means and standard deviations (SD) of the proprioceptive drift and questionnaire data (averaged across all items and split into its subcategories) in Experiment 1.

Conditions		Proprioceptive drift		Questionnaire data (-2 to +2)							
				average		Ownership		Agency		Location	
MF	TF	Mean	SD	Mean	SD	Mean	SD	Mean	SD	Mean	SD
s	s	7.00	3.26	0.11	0.93	-0.16	1.09	1.14	0.98	-0.25	1.12
a	s	4.00	3.27	-0.21	0.99	-0.47	1.15	0.74	1.19	-0.44	1.01
s	a	3.84	2.76	0.01	0.87	-0.31	0.98	1.05	0.98	-0.43	1.04
a	a	1.27	1.35	-0.42	0.86	-0.80	0.92	0.68	1.14	-0.59	0.98

MF = motor feedback; TF = tactile feedback; ‘s’ = synchronous; ‘a‘ = asynchronous.

**Fig 3:**
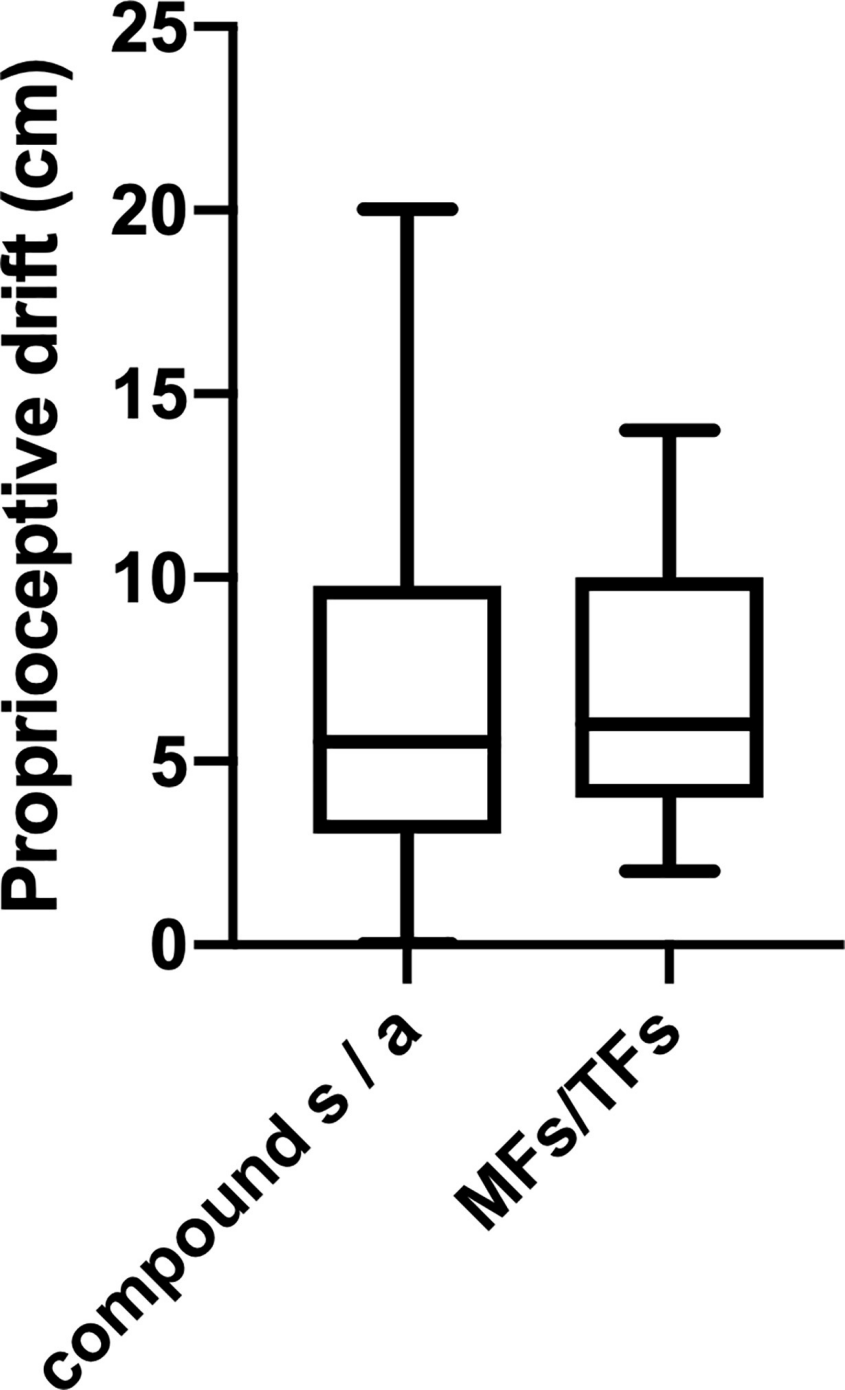
Box-plots for the means and standard deviations of the proprioceptive drift in the fully synchronous motor (MF) and tactile feedback (TF) condition (MFs/TFs), compared to the compound score, reflecting the linear combination of effects caused by synchronous (s) and asynchronous (a) feedback.

Additionally, more detailed post-hoc comparisons revealed significant differences between all conditions, except for the contrast testing *MFa/TFs* against *MFs/TFa* (Table 3), indicating that synchrony in one factor compensates for asynchrony in the other one. In order to test whether combined sensorimotor information under conditions of double synchrony, i.e., *MFs/TFs* (mean = 7.00 cm; SD = 3.26; Table 2), might have increment value for the recalibration of spatial body coordinates, we compared its effect on the proprioceptive drift with the compound score (mean = 6.57 cm, SD = 4.69) representing the summation of the effects in the mixed conditions. We found evidence against a difference between the two values (*BF*_10_ = 0.27), suggesting no enhancing effect caused by sensorimotor combination (Fig 3).

**Table 3:** Post hoc comparisons for the proprioceptive drift in Experiment 1. All *p*-values are Bonferroni-corrected.

Conditions		Mean Difference	SD	*t*_43_	*p*_corr_
**MFs/TFs**	MFa/TFs	3.00	2.06	9.67	< .001
	MFs/TFa	3.16	2.16	9.72	< .001
	MFa/TFa	5.73	2.55	14.92	< .001
**MFa/TFs**	MFs/TFa	0.16	2.20	0.48	1.000
	MFa/TFa	2.73	2.57	7.06	< .001
**MFs/TFa**	MFa/TFa	2.57	1.94	8.76	< .001

SD = standard deviation; MF = motor feedback; TF = tactile feedback; ‘s’ = synchronous; ‘a‘ = asynchronous.

#### Questionnaire data

In most conditions, the averaged questionnaire data (Table 2) did significantly differ from 0 in the positive direction (*t*_43_ between 0.84 and 1.40, *p*_corr_ between .676 and 1.000, Cohen’s *d* between -0.21 and 0.12), except for the *MFa/TFa* condition (*t*_43_ = -3.27, *p*_corr_ = .008, Cohen’s *d* = -0.49), with significantly negative ratings. By performing an ANOVA on the averaged data, we found a significant main effect for *MF* (*F*_1,43_ = 20.22, *p* < .001, η^*2*^ = .32), with post-hoc tests revealing higher (*p*_corr_ < .001) ratings in the synchronous compared to the asynchronous level, but no significant effect for *TF* (*F*_1,43_ = 2.03, *p* = .161, η^*2*^ = .05), and no significant interaction (*F*_1,43_ = 0.89, *p* = .350, η^*2*^ = .02). These results indicate that only synchronous visuomotor information in the RobHI induced significant, albeit weak embodiment experiences for the device. In a next step, we further analysed the factors of the questionnaire, i.e., *Ownership*, *Location*, and *Agency*. The ANOVA for *Ownership* revealed a significant main effect of *MF* (*F*_1,43_ = 12.71, *p* < .001, η^*2*^ = .23), with stronger ownership sensations in the synchronous compared to the asynchronous level (*p*_corr_ < .001), but no significant effect for *TF* (*F*_1,43_ = 3.19, *p* = .081, η^*2*^ = .07) or the interaction of both factors (*F*_1,43_ = 1.72, *p* = .197, η^*2*^ = .04). For *Agency*, we also found a significant main effect of *MF* (*F*_1,43_ = 12.94, *p* < .001, η^*2*^ = .23), with stronger agency sensations in the synchronous level (*p*_corr_ < .001), but no significant main effect for *TF* (*F*_1,43_ = 0.64, *p* = .427, η^*2*^ = .020) or the interaction of both factors (*F*_1,43_ = 0.03, *p* = .864, η^*2*^ = .001). Both results indicate that only the kind of motor feedback influence the intensity of robotic hand ownership and agency sensations. For *Location*, neither a main effect of *MF* (*F*_1,43_ = 2.87, *p* = .097, η^*2*^ = .06) and *TF* (*F*_1,43_ = 2.62, *p* = .113, η^*2*^ = .06) nor an interaction (*F*_1,43_ = 0.04, *p* = .835, η^*2*^ = .001) was found, suggesting differential effects on implicit, i.e., the proprioceptive drift, and explicit measures of hand position.

### Experiment 2

#### Proprioceptive drift

Means and standard deviations for the proprioceptive drift are given in Table 4. All of these mean values differed significantly from 0 in each condition (*t*_17_ between 4.74 and 10.59, all *p*_corr_ ≤ .001; Cohen’s *d* between 1.12 and 2.50), indicating a significant perceived drift towards the robotic hand.

**Table 4:** Means and standard deviations (SD) of the proprioceptive drift and questionnaire data (averaged across all items) in Experiment 2.

Conditions		Proprioceptive drift		Questionnaire data (-2 to +2)							
				average		Ownership		Agency		Location	
MF	TF	Mean	SD	Mean	SD	Mean	SD	Mean	SD	Mean	SD
s	s	9.01	3.61	-0.60	0.99	-0.60	0.99	0.19	1.50	-0.42	1.31
a	s	5.67	3.25	-0.66	0.96	-0.66	0.96	-0.19	1.38	-0.39	1.07
s	a	5.62	3.14	-0.84	0.74	0.84	0.74	0.06	1.50	-0.56	1.16
a	a	3.67	2.52	-1.08	1.10	-1.08	1.10	-0.06	1.45	-0.64	1.35
s	n	3.96	2.21	-1.02	0.91	-1.02	0.91	-0.22	1.43	-1.11	0.68
a	n	3.65	2.06	-1.53	0.60	-1.53	0.60	-0.39	1.38	-1.31	0.77
n	s	3.32	2.46	-0.77	0.95	-0.77	0.95	-0.72	1.13	-0.78	0.90
n	a	2.88	2.13	0.67	0.82	0.67	0.82	-0.78	1.07	-0.42	1.20
n	n	1.89	1.69	-1.53	0.56	-1.53	0.56	-1.42	0.81	0.94	1.22

MF = motor feedback; TF = tactile feedback; ‘s’ = synchronous; ‘a‘ = asynchronous; ‘n’ = no feedback. For illustrative purposes, the conditions are arranged by bimodal, unimodal, and absent sensorimotor input.

**Fig 4:**
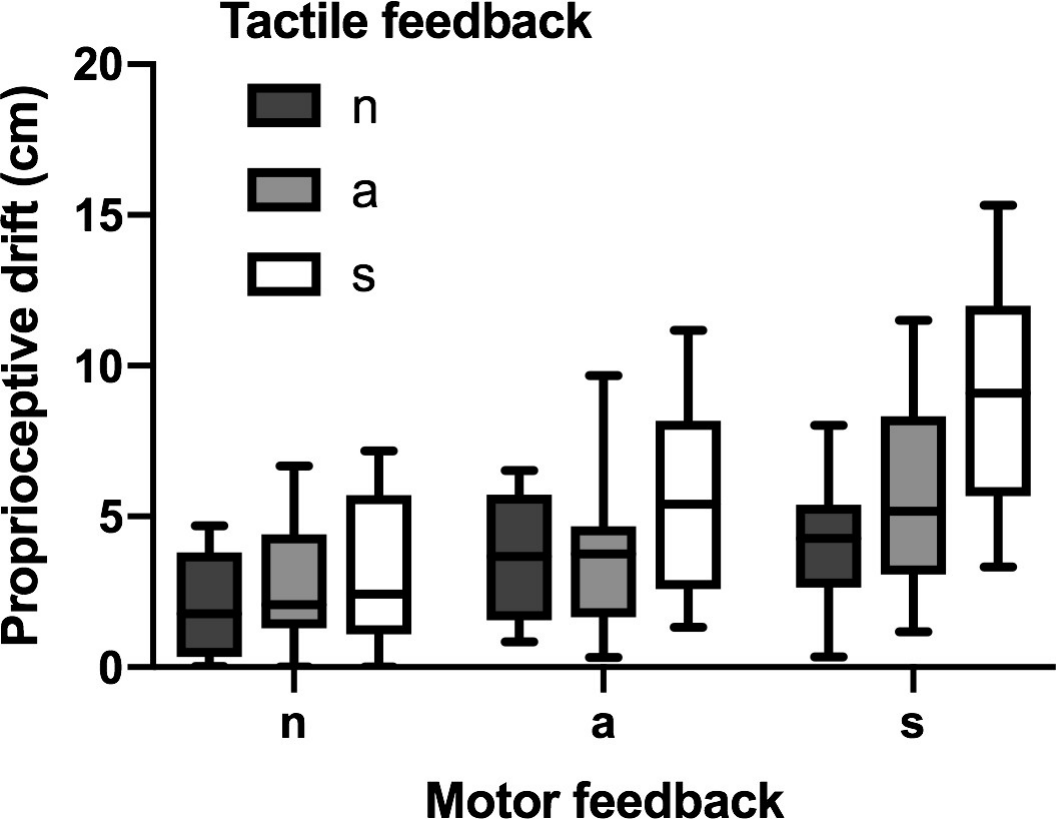
Proprioceptive drift under conditions of motor (MF) and tactile feedback (TF) with their levels synchronous (s), asynchronous (a), and no feedback (n).

The 3 (*MF*) × 3 (*TF*) repeated-measures ANOVA revealed significant main effects for both *MF* (*F*_1.51,25.59_ = 106.80, *p* < .001, η^*2*^ = .86) and *TF* (*F*_2,34_ = 69.87, *p* < .001, η^*2*^ = .80). Paired post-hoc tests contrasting the factor levels revealed that for both *MF* (*t*_17_ between 6.92 and 11.88, all *p*_corr_ < .001, Cohen’s *d* between 1.66 and 3.56) and *TF* (*t*_17_ between 4.14 and 9.50, all *p*_corr_ ≤ .002, Cohen’s *d* between 1.08 and 2.81) there was an identical order of *s* > *a* > *n*. This indicates that even asynchronous feedback is used at least partly as information source for the calibration of spatial body coordinates. The interaction between both factors became significant (*F*_2.84,48.19_ = 20.34, *p* < .001, η^*2*^ = .55), which is illustrated by Fig 4.

Additionally, we performed more detailed post-hoc comparisons for the crucial 2 (*TFs/TFa*) × 2 (*MFs/MFa*) combination of conditions, i.e., the replication of the findings reported for Experiment 1, as well as for the 2 (*TFs/TFn*) × 2 (*MFs/MFn*) combination of conditions, which serve to further evaluate the relative contribution of visuotactile and visuomotor input to the calibration of body-space coordinates. The results again indicate that synchrony or presence, respectively, in one factor can compensate for asynchrony or absence, respectively, of the other one (note the non-significant contrasts for the mixed conditions in Table 5 and Table 6, respectively).

**Table 5:** Post-hoc paired *t*-tests (two-tailed) for the proprioceptive drift in Experiment 2 (detailed testing of synchronous (s) and asynchronous (a) conditions). All *p*-values are Bonferroni-corrected.

Conditions		Mean Difference	SD	*t*_17_	*p*_corr_
***MFs/TFs***	*MFa/TFs*	3.34	1.30	10.93	< .001
	*MFs/TFa*	3.40	1.80	8.00	< .001
	*MFa/TFa*	5.33	1.74	13.03	< .001
***MFa/TFs***	*MFs/TFa*	0.05	1.25	0.16	1.000
	*MFa/TFa*	1.99	1.51	5.59	< .001
***MFs/TFa***	*MFa/TFa*	1.94	1.75	4.72	.001

SD = standard deviation; MF = motor feedback; TF = tactile feedback.

**Table 6:** Post-hoc paired *t*-tests (two-tailed) for the proprioceptive drift in Experiment 2 (detailed testing of combined synchronous (s) and no-feedback (n) conditions). All *p*-values are Bonferroni-corrected.

Conditions		Mean Difference	SD	*t*_17_	*p*_*corr*_
***MFs/TFs***	*MFn/TFs*	5.05	1.80	11.89	< .001
	*MFS/TFn*	5.69	1.66	14.52	< .001
	*MFn/TFn*	7.12	2.28	13.26	< .001
***MFn/TFs***	*MFs/TFn*	0.65	1.13	2.44	.156
	*MFn/TFn*	2.07	1.46	6.05	< .001
***MFs/TFn***	*MFn/TFn*	1.44	1.21	4.98	< .001

SD = standard deviation; MF = motor feedback; TF = tactile feedback.

**Fig 5:**
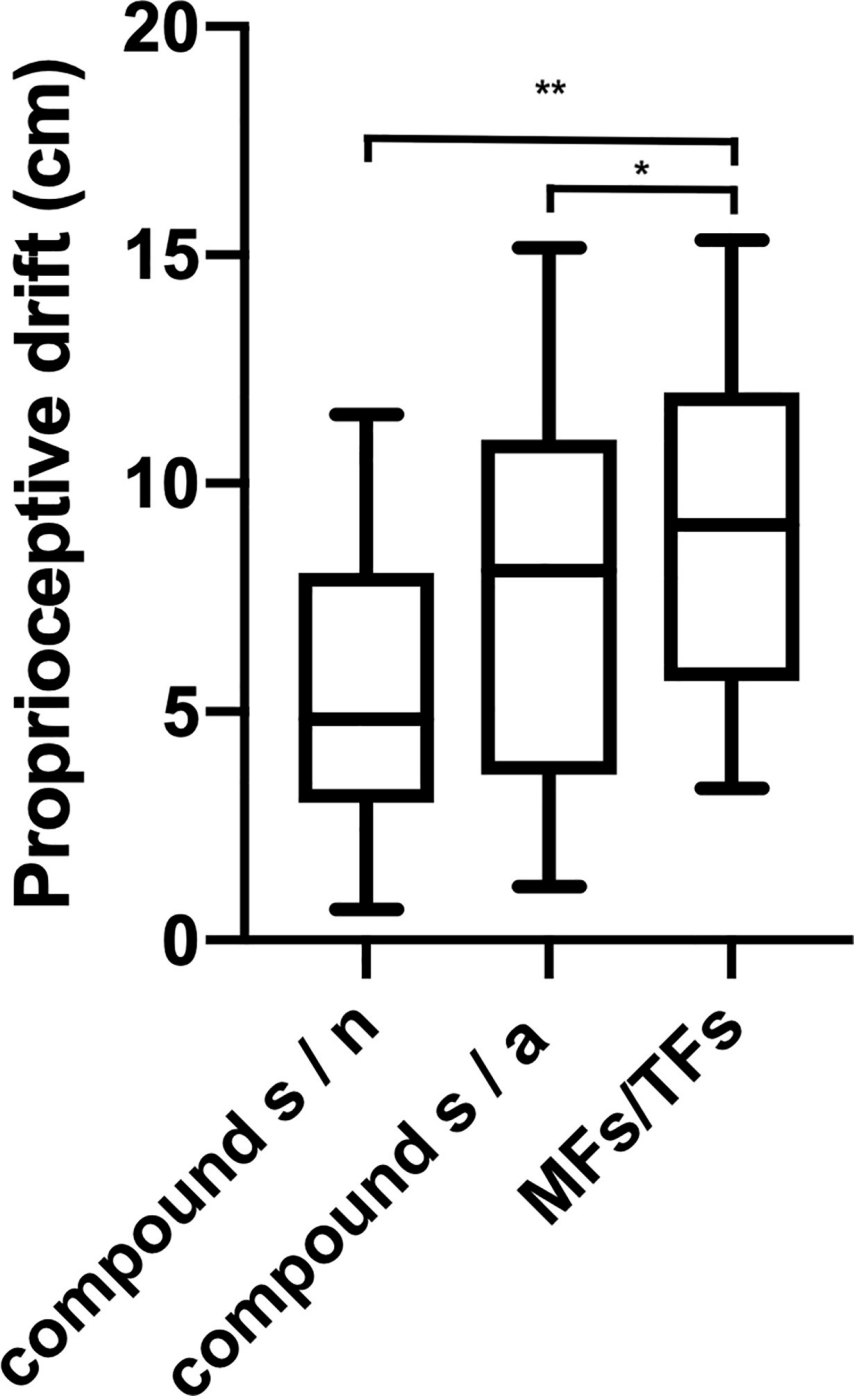
**Box-plots for the means and standard deviations of the proprioceptive drift in the fully synchronous motor (MF) and tactile feedback (TF) condition (MFs/TFs), compared to a) the compound score s/a, reflecting the linear combination of effects caused by synchronous (s) and asynchronous (a) feedback, and to b) the compound score s/n, reflecting the linear combination of effects caused by unimodal feedback.** Bayes factors (BF): * BF_10_ < 3; **BF_10_ >100.

Again, we tested whether combined sensorimotor information under conditions of double synchrony, i.e., *MFs/TFs* (mean = 9.01 cm, SD = 3.61; Table 4), might have increment value for the recalibration of spatial body coordinates compared to the compound score for mixed *s/a* conditions (mean = 7.61 cm, SD = 4.22) and to the compound score for mixed *s/n* conditions (mean = 5.39 cm, SD = 3.22). We only found weak evidence for an enhanced effect caused by sensorimotor combination in the first case (*BF*_10_ = 2.63), but strong evidence for an enhanced combinatory effect in the second case (*BF*_10_ > 100). This effect is visualized in **Fig 5**.

#### Questionnaire data

Means and standard deviations of the average total questionnaire scores are given in Table 4. The analysis revealed no significant main effect for *MF* (*F*_1.35,22.98_ = 2.80, *p* = .098, η^*2*^ = .14), but a significant effect for *TF* (*F*_1.39,23.54_ = 14.24, *p* < .001, η^*2*^ = .46). Paired post-hoc tests contrasting the factor levels revealed *TFs* = *TFa* (t_*17*_ = 0.95, *p*_corr_ = 1.000, Cohen’s *d* = 0.46) and the order *s* = *a* > *n* (t_*17*_ = 3.89 and 4.89, both *p*_corr_ < .001, Cohen’s *d* = 1.89 and 2.37, for the *t*-tests comparing *s* and *n* or *a* and *n* factor levels). The interaction was not significant (*F*_3.15,53.60_ = 0.51, *p* = .689, η^*2*^ = .03).

## Discussion

In two experiments, we used a sensor glove-controlled robotic hand equipped with pressure sensors for providing vibrotactile feedback at participants’ fingertips in a robotic hand illusion (RobHI) paradigm. This human-in-the-loop approach enabled us to independently control visuomotor and visuotactile feedback in terms of synchrony between actual movement/stimulation and the respective feedback as well as disabling each feedback individually. We assessed the perceived location of the own hand explicitly (by using a questionnaire) and implicitly (by the induced proprioceptive drift) as well as the subjective experience of ownership and agency for the robotic hand. In Experiment 1, we found that synchronous motor feedback as well as synchronous tactile feedback induced significantly higher proprioceptive drifts towards the robotic hand compared to the other conditions, while only synchronous motor feedback was associated with significantly more intense ownership and agency sensations. Crucially, for the calibration of spatial body coordinates, we found that synchrony in one kind of feedback appears to compensate for the asynchrony in the other modality, with equally contributing effects. In Experiment 2, we replicated most of these findings and complemented them by the implementation of conditions in which we manipulated the presence/absence of both modalities. These results suggest that the presence of both modalities, compared to only one of them, results in a super-additive effect on the calibration of body-space coordinates. These results highlight that both motor efferents and multimodal sensory input are conducive to the coding of the body in space and substantiates the extended methodical capabilities that emerge by using experimental designs including robotic limbs. The compensatory effects found in Experiment 1 and 2 and the super-additive effect by the presence of both modalities in Experiment 2 furthermore justifies our assumption of an enhancing effect caused by sensorimotor combination and integration on body-space representation. In contrast to other studies (e.g., [25]), we used a within-subject design in order to reduce inter-individual variation and thus were able to detect also small interactions between visuomotor and visuotactile information. Furthermore, we extended the results reported by Padilla-Castañeda et al. [19], since we modulated the synchrony of motor feedback instead of comparing active with passive conditions. To our knowledge, this study is therefore the first one that investigates the effects of both visuomotor and visuotactile feedback on body-space representation in a within-subject design.

The results of this study are in accordance with previous studies regarding the elicitation of the RHI under synchronous and asynchronous stimulation conditions (e.g., [7, 32–34]). When testing for significant differences between the experimentally induced and baseline drift values, we found that all conditions, in both experiments, were significantly different from the baseline. This finding is in accordance with the results reported in earlier studies (e.g., [34]) which have shown that proprioceptive drift occurs even in asynchronous stimulation conditions as long as a visuoproprioceptive conflict is induced.

Furthermore, our findings replicate and extend the results of Romano et al. [35] who demonstrated that the elicitation of the RobHI (by measuring the proprioceptive drift) primarily depends on visuomotor correlations. We could show in addition that the calibration of spatial body coordinates does not only rely on visual and motor feedback but depends on the synchrony of both visuomotor and visuotactile feedback in a combinatory fashion. Interestingly, contrary to the present study, Romano et al. [36] admittedly found an effect for the proprioceptive drift, but not for explicit robotic hand embodiment experiences such as ownership or agency. This inconsistency of findings might rely on the different mode of control in both studies: while we used a sensor glove, Romano et al. [35] controlled their robotic hand by electromyographic signals. However, also in the present study, the findings are rather inconsistent since we found contradictory results for MF or TF contribution on conscious body perception in Experiment 1 and 2.

It is remarkable, however, that the largest effects in both our and the Romano et al. [35] study were found for the proprioceptive drift. Although we found significantly stronger ownership and agency experiences associated with synchronous motor feedback, the effects were rather small compared to the behavioral drift effects, and participants predominantly negated having these sensations, as indicated by negative ratings. Previous studies argued that negative Likert ratings can still be indicators of an illusion experience, as long as there is a relative difference to control conditions (e.g., [36]). Yet, it is important to note that the ratings are much lower than in previous studies using visuotactile or visuomotor stimulation for the induction of bodily illusions (e.g., [13, 16]). This might be explained by deviations between the appearance of the robotic hand and a human hand [37] or differences in the size between the robot hand and the participants’ hand [38]. Hence, there is potential for technical improvements in future studies and replications based on the RobHI. Besides the above-mentioned appearance of the robotic hand, the cable-driven flexing motions of the fingers might be optimized and a more durable design would reduce calibration efforts and improve overall stability. Another possibility for improvement might be a more realistic way of holding the rubber ball in place. Finally, robotic hands that better match individual characteristics of the participants’ hand would make an improvement. It remains to be noted that more research is needed to elucidate the complex multimodal interactions on conscious body ownership.

Contrary to relatively low or even absent effects on conscious body perception, we found strong effects for the proprioceptive drift. This indicates that the induced shift in body-space representation by the RobHI might rely more on stimulation conditions than on the induction of illusory embodiment, supporting previous results [34]. Furthermore, there might be some kind of dissociation between explicit and implicit spatial representations of one’s own body. The results of the present study indicate that the interplay of visuotactile and visuomotor congruency contribute to the recalibration of body coordinates in peripersonal space in independent ways. Interestingly, our data indicate that the presence of asynchronous feedback has still stronger effects than the complete absence of this modality. Asynchrony in both sensory and motor feedback has been earlier associated with a diminished effect of illusory setups. This is also true for our data. However, if compared to no feedback, asynchronous feedback actually seems to have some kind of informative value for the recalibration of body-space representation, which is superior to the absence of any feedback. This appears to be especially true for motor feedback, which might be experienced as relying on one’s own movements as long as the delay in visual feedback is fixed. Prospective studies still have to investigate how asynchronous feedback is resolved and used for the representation of the body in space.

The compensatory and super-additive effects we found in our study might rely on identical neural underpinnings. Previous neuroscientific research has revealed some crucial mechanisms underlying sensory integration and multisensory enhancement, the latter one describing a greater neural response to multisensory input compared to both unisensory contributions [39]. The superior colliculi, which are located in the mammalian midbrain and are involved in the control of eye and head movements based on multimodal sensory input, have been shown to play an important role in the enhancing effect of multisensory integration [40]. Interestingly, it has been earlier proposed that these regions and their functions might also be relevant for the processing of bodily self-consciousness [41]. A recent study on the neural underpinnings of the RHI showed illusion-associated increased functional connectivity between the superior colliculi and other brain areas of the body network such as the temporoparietal junction and the premotor cortex [42] which also code for the location of the body in space (e.g., [43, 44]). Our finding of a super-additive effect of multimodal input on proprioceptive drift might reflect a behavioral correlate of these neural processes.

Our results align with the understanding of human sensorimotor systems as Bayesian ideal observers (e.g., [45, 46]). Following that reasoning, the integration of multiple uncertain sources of information can be assumed to generally produce more precise estimates than each individual source of information. Accordingly, the behavior observed in our experiment could be expected under the assumption that humans employ some form of optimal cue integration, similar to Bayesian ideal observer models. Recently, a Bayesian account has been applied to body perception as well [47] insofar that the brain might use predictive coding strategies for bodily self-recognition. Multisensory recalibration of body-space coordinates, even by asynchronous input as reported in the present study, might help to better understand the involved processes.

The present results might have important implications for the design of human-machine interfaces and assistive robotics such as prostheses. Recently, it has been shown that prostheses change peripersonal space representation in amputees [48] and it has been suggested that prostheses, which are perceived as being a part of the body, are even more capable in modulating the amputees body-space representation [49]. Arm prostheses which are embodied by their users are more capable to stabilize body posture than unembodied prostheses [50]. Moreover, prostheses, which are perceived as belonging to the amputee’s body, are associated with lower levels of phantom limb pain [51], a common consequence of limb amputation. All these findings suggest that eliciting embodiment sensations for prostheses might not only shape body-space representation, but also have important therapeutic implications [52]. Moreover, closed-loop user control is currently seen as an important design goal in prosthetic engineering [53, 54], which is supported by the present study due to the bimodal and synchronous conditions achieving the strongest results. Although we implemented controllability for the robotic hand with a sensor glove in the present study, which is not appropriate for amputees due to obvious reasons, our results still have important implications for prospective prosthetic developments. Thus, tactile feedback might not only facilitate interaction with the environment [55], and modulate perceived ownership for the prosthetic limb [56, 57], but also shape peripersonal space representation, especially when combined with myoelectric control systems. All these positive effects might enhance the amputees’ motivation to use the prosthesis, reduce the risk of its rejection, and thus contribute to increase the patients’ competence in daily life. The human-in-the-loop approach can further be extended by varying the implementation of feedback to investigate their potential for human-machine interface designs (e.g., altering the modality or location of feedback). In the long run, these methods and the corresponding results thus have high potential to guide the design of assistive robotic devices such as prosthetics or exoskeletons [55].

It bears noting that the present study has several limitations, which might have implications for methodological improvements in future studies. Firstly, although the intrinsic delays of the RobHI system (about 80 to 120 ms) are below the temporal asynchrony values abolishing illusory body experiences identified in previous studies (about 300 ms [9, 18, 26, 27]), even those slight delays might have interfered with the RobHI. Future studies might take advantage of technological improvements for reducing system-intrinsic delays in combination with approaches for establishing individual thresholds, for instance by temporal order or simultaneity judgment tasks [58]. Prospective studies could also take advantage of more elaborated approaches for assessing the perceived location of the participants’ own hand, including those that record participants’ answers electronically (e.g., [59]). Furthermore, recent results indicate that the drift phenomenon associated with the RHI is rather complex and involves body representations of different modalities [59, 60]. Thus, it might be advisable to use more implicit measures of the dynamics peripersonal space boundaries, as recently developed [61].

## Conclusions

The results of this study suggest an equal contribution of visuotactile and visuomotor feedback to implicit measures of spatial body representation. A combination of appropriate multimodal feedback can be used to not even compensate for inappropriate feedback, but also enhance this effect, probably due to facilitating processes of sensorimotor combination and integration. One particularly intriguing finding is that conflicting bimodal cues entailed a stronger effect on body-space coordinates than unimodal synchronous ones. The results further demonstrate that the strength of explicit measures of robotic hand embodiment depends on a complex interplay between different kinds of multimodal feedback, which might have implications for clinical applications as well.

## Supporting information

S1 File Archive containing acquired raw data (questionnaire scores and proprioceptive drift) for each experiment. (ZIP)
